# Health Care Access Outcomes for Immigrant Children and State Insurance Policy

**DOI:** 10.1001/jamanetworkopen.2025.45826

**Published:** 2025-12-01

**Authors:** Katherine E. Douglas, Michael C. Monuteaux, Katherine R. Peeler, Ananya Tadikonda, Catherine G. Coughlin, Julie M. Linton, Lois K. Lee

**Affiliations:** 1 Divison of Emergency Medicine, Boston Children’s Hospital, Boston, Massachusetts; 2 Divison of Medical Critical Care, Boston Children’s Hospital, Boston, Massachusetts; 3 Harvard Medical School, Boston, Massachusetts; 4 Department of Pediatrics, University of South Carolina School of Medicine Greenville, Greenville

## Abstract

**Importance:**

State policies provide variable health insurance coverage for the 3 million immigrant children in the US, with limited understanding of associations with health care access.

**Objectives:**

To (1) examine disparities in health insurance and health care access between immigrant and US-born children and (2) analyze associations of state insurance policies with health care access outcomes for immigrant children.

**Design, Setting, and Participants:**

This population-based, retrospective cross-sectional study sampled from the National Survey of Children’s Health (2016 to 2022) to facilitate national estimates. Caregivers of children aged 0 to 17 years completed the survey. Data were analyzed from May to December 2024.

**Exposures:**

Child immigration status and state-level health insurance policies for immigrant children, categorized as least inclusive (5-year waiting period and eligibility restricted by immigration status), moderately inclusive (waived 5-year waiting period), and most inclusive (waived 5-year waiting period and all immigrant children eligible).

**Main Outcomes and Measures:**

Primary outcomes were (1) uninterrupted health insurance in the past 12 months, (2) usual place for primary care, (3) usual place for sick care, (4) having foregone medical care, and (5) having difficulty with referrals. For each outcome, multivariable logistic regression models examined the association with immigration status, adjusting for state-level policies and individual variables, reporting adjusted odds ratios (aORs) and 95% CIs.

**Results:**

There were 277 386 children included (population estimate, 72 473 052 children) with 8835 immigrant children (population estimate, 3 097 329 children [4.3%]; 1 513 509 [48.9%] aged 12-17 years; 1 542 412 female [49.8%]) and 268 551 US-born children (population estimate, 69 375 723 children [95.7%]; 23 450 439 [33.8%] aged 12-17 years; 33 876 023 female [48.8%]). In multivariable analyses, immigrant compared with US-born children had lower odds of uninterrupted health insurance (aOR, 0.48; 95% CI, 0.41-0.56), usual place for primary care (aOR, 0.44; 95% CI, 0.38-0.52), and usual place for sick care (aOR, 0.62; 95% CI, 0.55-0.70), and increased odds of having foregone medical care (aOR, 1.61; 95% CI, 1.22-2.14) and difficulty with subspecialty referral (aOR, 1.54; 95% CI, 1.16-2.04). Immigrant children in the most compared with least inclusive policy states had increased odds of uninterrupted health insurance (aOR, 3.01; 95% CI, 1.89-4.79) and usual place for primary care (aOR, 1.61; 95% CI, 1.07-2.41).

**Conclusions and Relevance:**

In this cross-sectional study of US children, immigrant compared with US-born children had disparities in health care access, which were attenuated in states with the most inclusive state insurance policies, suggesting that inclusive state insurance eligibility policies for immigrant children may improve health care access outcomes for this population.

## Key Points

**Question:**

Are there associations of state-level policies for insurance eligibility for immigrant children with improved health care access outcomes for this population?

**Findings:**

In this cross-sectional study of 277 386 children, immigrant compared with US-born children had significantly worse outcomes for uninterrupted health insurance, access to primary care, access to sick care, having foregone medical care, and having difficulty with referrals. More inclusive state-level insurance eligibility policies for immigrant children were associated with improved access to health insurance and primary care.

**Meaning:**

These findings suggest that more inclusive state insurance eligibility policies for immigrant children may improve health care access outcomes for this population.

## Teaser

This cross-sectional study examines disparities in health insurance and health care access between immigrant and US-born children and analyzes associations of state insurance policies with health care access outcomes for immigrant children.

## Introduction

Immigrants, born outside of the US, represented an estimated 3 million children in 2021.^1^ There is inequitable health insurance and health care access for immigrant children compared with US-born children.^2^ Uninsured children in immigrant families are more likely to lack a consistent source of care and have unmet health care needs.^3^

Public insurance access for immigrant children has been limited, impacting health care access. In most states, only lawfully present immigrants who meet program requirements for income and state residency may enroll in Medicaid and the Children’s Health Insurance Program (CHIP). This initially required holding a qualified immigration status for at least 5 years. Since the passage of the CHIP reauthorization act (CHIPRA) in 2009, 36 states and the District of Columbia have taken up a waiver to provide coverage to lawfully residing children without the 5-year waiting period. Furthermore, as of January 2025, 14 states and the District of Columbia provide insurance coverage for all children regardless of immigration status.^4^

On a national level, few studies have explored how differences in state policies for health insurance eligibility for immigrant children may impact insurance coverage and health care access outcomes. These studies included populations of immigrant children prior to 2020 and overall found improved health care access outcomes for children in states with expanded insurance eligibility.^5,6^ Additionally, policies not directly related to health insurance access, such as policies facilitating obtaining a driver’s license regardless of immigration status, have been associated with increased access to health care for immigrant chldren.^7^

There is a gap in understanding contemporary national-level disparities and the impacts of variable state health insurance eligibility policies for immigrant compared with US-born children for health insurance and health care access. The objectives of this study are to examine disparities in health insurance and health care access for immigrant compared with US-born children and the association of state health insurance eligibility policies for immigrant children with these outcomes. We hypothesized that, among immigrant children, living in states with more inclusive policies for health insurance eligibility would be associated with improved health care access outcomes.

## Methods

### Study Design, Population, and Data Sources

We performed a retrospective, cross-sectional study using the National Survey of Children’s Health (NSCH), sponsored by the Maternal and Child Health Bureau as part of the US Department of Health and Human Services.^8^ NSCH surveys caregivers of children aged 0 to 17 years on measures of health, health care access, and demographic and neighborhood characteristics. Responses are weighted to account for nonresponse and to reflect the US population. We used data from 2016 to 2022, and all caregiver survey responses were included in the study. Participants were excluded if there was missing data for the primary outcomes or covariates. This study followed the Strengthening the Reporting of Observational Studies in Epidemiology (STROBE) reporting guideline and was deemed exempt from review and the requirement of informed consent by the Boston Children’s Hospital institutional review board because data were publicly available and deidentified.

### Study Measures

The primary outcomes were (1) uninterrupted insurance in past 12 months; (2) having a usual place of primary care; (3) having a usual place for sick care that is not a hospital emergency department, consistent with NSCH definition of usual place for sick care^8^; (4) foregone health care; and (5) difficulty with subspecialty referrals (eTable 1 in Supplement 1). The primary exposure was child immigration status, defined by the survey question, “Was this child born in the United States?” For the secondary exposure of state insurance eligibility policies, states were classified as (1) least inclusive (only certain immigration statuses qualify for insurance and there is a a required 5-year waiting period), (2) moderately inclusive (only certain immigration statuses qualify for insurance and no 5-year waiting period), and (3) most inclusive (insurance access for all children regardless of immigration status and no 5-year waiting period).^5,6^ The categories of child race and ethnicity were caregiver-reported and were included as proxies for structural racism and its impact on access to health care. We included the NSCH race categories of American Indian or Alaska Native, Asian, Black or African American, Native Hawaiian and Other Pacific Islander, White, and other (including 2 or more races) and ethnicity categories of Hispanic or Latino origin or not Hispanic or Latino origin. Race and ethnicity were reported as a single variable in accordance with US Census Bureau recommendations.^9^

We also included an additional variable of state driver’s license policies, which allows individuals to obtain a driver’s license regardless of immigration status because this could be a proxy for transportation access for health care.^7,10^ State-level policy data were obtained from the Urban Institute^10^ and confirmed via review of state legislation through state websites (eTable 2 in Supplement 1). All state-level variables were measured annually to account for within-state variation over time.

### Statistical Analysis

We calculated frequencies of demographic characteristics for immigrant and US-born children and their caregivers. Then we compared the health insurance and health care access outcomes between these 2 groups. Among the subgroup of immigrant children, we compared child insurance status (categorized as public insurance, private insurance, no insurance, or insured with gaps in coverage) between groups defined by inclusivity of state-level insurance policy (ie, least inclusive, moderately inclusive, and most inclusive).

We estimated a series of multivariable logistic regression models, with each of the outcomes as the dependent variable. Models included the primary (immigration status) and secondary (state-level health insurance inclusivity, modeled as indicator variables with least inclusive set as the referent) exposures as independent variables. All models were adjusted for individual-level child characteristics (age, sex, and race and ethnicity) and caregiver characteristics (language used for survey, immigration status, highest education, employment status, and household income, categorized by percent of the Federal poverty level [FPL]). FPL is imputed in 15% to 20% of cases in NSCH and we imputed this value in each multivariable model using the 6 imputations provided by NSCH.^11^ Based on previous research, we also adjusted for state-level factors in the multivariable models including state median income (from the US Census Bureau)^12^ and driver’s licensing based on immigration status.^7,10^ As stated previously, state-level variables, including policies, were modeled as time-varying covariates (ie, measured annually within state). Observations with missing data on any variable included in the model (except FPL) were omitted from the analysis for a complete case analysis. Models reported adjusted odds ratios (aORs) with 95% CI.

We then performed subgroup analyses for all outcomes with the population of immigrant children. We created an interaction term between child immigration status and state insurance law inclusivity to further explore this association. We also performed a sensitivity analysis using multiple imputation (6 versions to match NSCH imputations) to account for missing data in our multivariable models. We analyzed the relative variance increase and the fraction of missing information to evaluate the impact of missingness on variance estimation and the adequacy of the number of imputations, respectively.^13^ All analyses were conducted within the context of the survey design characteristics of NSCH, using the child as the unit of analysis. We specified the primary sampling units, patient visit sampling weights, and the stratum identifiers as implemented in the STATA svyset command, allowing for the calculation of nationally-representative estimates. We conducted all analyses from May to December 2024 in STATA 16.0 (StataCorp).^14^ Statistical significance was defined as a 2-sided *P* < .05.

## Results

The starting sample consisted of 279 546 children. Of these, 277 386 children (99.2%) had valid immigration status data, comprising the study sample, representing a population estimate of 72 473 052 children. Of the study sample, 8835 children (3.2%) were classified as immigrant children, representing 3 097 329 children nationally (4.3%), and 268 551 children (96.8%) were classified as US-born, representing 69 375 723 children (95.7%) nationally. Among immigrant children 1 513 509 (48.9%) were 12 to 17 years, 1 542 412 (49.8%) were female, and 1 232 427 (39.8%) identified as Hispanic ethnicity. For race, 609 329 (19.7%) identified as non-Hispanic Asian, 510 582 (16.5%) as non-Hispanic Black, and 581 622 (18.8%) as non-Hispanic White. Among US-born children, 23 450 439 (33.8%) were 12 to 17 years, 33 876 023 (48.8%) were female, and 17 259 309 (24.9%) identified as Hispanic ethnicity. For race, 2 750 570 (4.0%) identified as non-Hispanic Asian, 8 949 168 (12,9%) as non-Hispanic Black, and 35 867 169 (51.7%) as non-Hispanic White. Immigrant compared with US-born children had higher proportions of being in the lowest income category (0%-199% FPL: 1 686 240 children [54.4%] vs 27 750 233 children [40.0%]) and of caregiver unemployment (522 573 children [17.6%] vs 7 459 291 children [11.1%]). Survey language for immigrant children included English (1 351 814 children [44.1%]) and Spanish (963 214 children [31.4%]), with 753 441 (24.6%) speaking other languages (Table 1).

**Table 1.** Demographics of Children in the U.S, National Survey of Children’s Health, 2016-2022^a^

Characteristic	Children, No. (%) (N = 279 546)		Population estimates, No. (%) (N= 72 473 052)	
	Immigrant children (n = 8835)	Born in the US (n = 268 551)	Immigrant children (n = 3 097 329)	Born in the US (n = 69 375 723)
State-level insurance law inclusivity				
Least inclusive	2299 (26.0)	86 478 (32.2)	556 444 (18.0)	16 926 231 (24.4)
Moderately inclusive	4490 (50.8)	78 939 (29.4)	1 611 841 (52.0)	33 766 997 (48.7)
Most inclusive	2046 (23.2)	46 983 (17.5)	929 045 (30.0)	18 682 495 (26.9)
Has state policy allowing driver’s license for immigrants	3530 (40.0)	86 039 (32.0)	1 042 138 (33.6)	21 512 290 (31.0)
Age, y				
0-5	1314 (14.9)	88 089 (32.8)	494 644 (16.0)	22 684 810 (32.7)
6-11	2752 (31.1)	78 939 (29.4)	1 089 176 (35.2)	23 240 474 (33.5)
12-17	4769 (54.0)	101 523 (37.8)	1 513 509 (48.9)	23 450 439 (33.8)
Sex				
Female	4467 (50.6)	129 473 (48.2)	1 542 412 (49.8)	33 876 023 (48.8)
Male	4368 (49.4)	139 078 (51.8)	1 554 918 (50.2)	35 499 700 (51.2)
Race and ethnicity (combined)				
Hispanic	2192 (24.8)	33 680 (12.5)	1 232 427 (39.8)	17 259 309 (24.9)
Non-Hispanic American Indian or Alaska Native	17 (0.2)	1605 (0.6)	3714 (0.1)	292 578 (0.4)
Non-Hispanic Asian	2898 (32.8)	12 137 (4.5)	609 329 (19.7)	2 750 570 (4.0)
Non-Hispanic Black or African American	848 (9.6)	16 499 (6.1)	510 582 (16.5)	8 949 168 (12.9)
Non-Hispanic Native Hawaiian and Other Pacific Islander	76 (0.9)	652 (0.2)	17 686 (0.6)	110 147 (0.2)
Non-Hispanic White	2256 (25.5)	184 766 (68.8)	581 622 (18.8)	35 867 169 (51.7)
Other^b^	548 (6.2)	19 212 (7.2)	141 969 (4.6)	4 146 782 (6.0)
Health insurance for child				
Public insurance	1903 (22.0)	53 698 (20.2)	905 840 (30.0)	19 997 313 (29.3)
Private insurance	5634 (65.2)	196 172 (73.9)	1 474 037 (48.8)	42 475 977 (62.2)
Unspecified	22 (0.3)	485 (0.2)	10 770 (0.4)	152 504 (0.2)
No insurance	787 (9.1)	9367 (3.5)	499 215 (16.5)	3 568 992 (5.2)
Insured but with gaps	299 (3.5)	5878 (2.2)	128 466 (4.3)	2 121 396 (3.1)
SHCN status				
SHCN	1733 (19.6)	62 391 (23.2)	462 587 (14.9)	13 557 759 (19.5)
Non-SHCN	7102 (80.4)	206 160 (76.8)	2 634 742 (85.1)	55 817 964 (80.5)
Caregiver income, % federal poverty level				
0-199	3343 (37.8)	74 191 (27.6)	1 686 240 (54.4)	27 750 233 (40.0)
200-299	1342 (15.2)	43 039 (16.0)	455 655 (14.7)	11 244 592 (16.2)
300-399	1002 (11.3)	38 962 (14.5)	231 333 (7.5)	8 413 800 (12.1)
≥400	3148 (35.6)	112 359 (41.8)	724 102 (23.4)	21 967 098 (31.7)
Caregiver employment status				
At least 1 caregiver employed at least part time	7442 (86.9)	240 404 (91.7)	2 448 947 (82.4)	59 703 622 (88.9)
No caregiver employed at least part time	1120 (13.1)	21 722 (8.3)	522 573 (17.6)	7 459 291 (11.1)
Caregiver highest education				
Less than high school	562 (6.4)	6312 (2.4)	562 890 (18.2)	6 050 307 (8.8)
High school	1072 (12.2)	34 460 (12.9)	568 821 (18.4)	13 291 576 (19.3)
Some college or associate degree	1333 (15.2)	60 133 (22.5)	438 652 (14.2)	14 872 586 (21.5)
College degree or higher	5827 (66.3)	166 570 (62.3)	1 517 179 (49.1)	34 823 808 (50.4)
Caregiver immigration status				
At least 1 caregiver born outside US	6040 (70.1)	45 013 (17.1)	2 448 696 (82.0)	17 231 784 (25.5)
No caregiver born outside US	2580 (29.9)	218 039 (82.9)	538 423 (18.0)	50 251 660 (74.5)
Language for survey				
English	5189 (59.1)	250 614 (93.8)	1 351 814 (44.1)	59 967 748 (87.1)
Spanish	1411 (16.1)	8588 (3.2)	963 214 (31.4)	6 213 314 (9.0)
Other	2173 (24.8)	7947 (3.0)	753 441 (24.6)	2 652 010 (3.9)

Abbreviation: SHCN, special health care needs.

^a^ Missing data accounted for 7% of total data (2% caregiver immigration status; 2% caregiver employment; 1% insurance type of the child; and <1% for other variables).

^b^ Two or more races or any race or ethnicity not otherwise specified.

**Figure 1.**
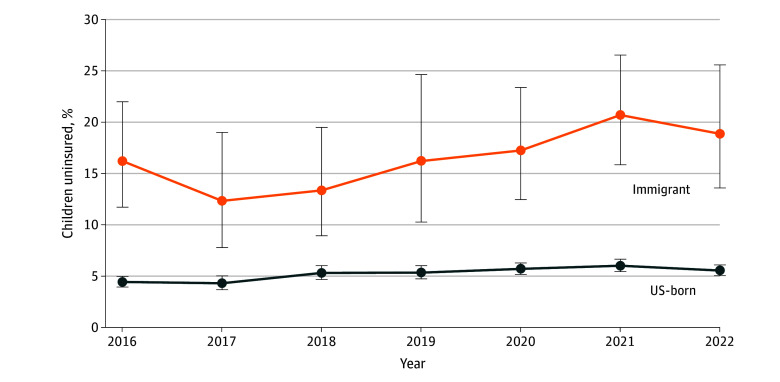
Uninsurance Rates by Immigration Status, National Child Health Survey, 2016-2022 Figure shows uninsurance rates of immigrant and US-born children over the 7-year study period, with vertical lines representing 95% CIs.

**Figure 2.**
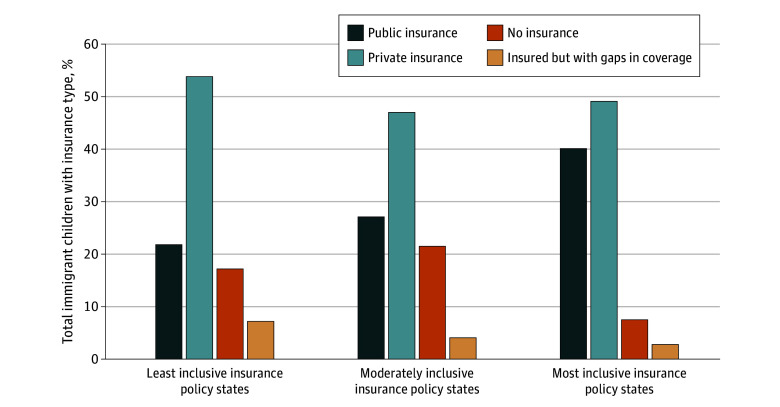
State Insurance Law Inclusivity and Insurance Status for Immigrant Children Figure demonstrating insurance status in the past 12 months for immigrant children, divided by the inclusivity of insurance policy for the state the child lives in.

For the primary outcome of health insurance access, 499 215 immigrant children (16.5%) were uninsured during the entire past 12 months compared with 3 568 992 US-born children (5.2%). Gaps in health insurance coverage occurred among 128 466 immigrant children (4.3%) compared with 2 121 396 US-born children (3.1%) (Table 1). Uninsurance rates varied over the study period, with higher rates for immigrant children across all years, including more than 20% uninsured in 2021 (Figure 1). Immigrant compared with US-born children reported worse health care access outcomes with higher rates of having no primary care, no usual place for sick care, having foregone medical care, and having difficulty with subspecialty referral (eTable 3 in Supplement 1). Among immigrant children, there were differences in insurance status composition for those in the most inclusive compared with least inclusive and moderately inclusive insurance policy states (Figure 2).

In the multivariable analysis including the study population with complete data, immigrant compared with US-born children had lower odds of uninterrupted health insurance (aOR, 0.48; 95% CI, 0.41-0.56), usual place for primary care (aOR, 0.44; 95% CI 0.38-0.52), and usual place for sick care (aOR, 0.62; 95% CI, 0.55-0.70), after adjusting for state policies and child and caregiver factors. Immigrant compared with US-born children also had higher odds of having foregone medical care (aOR, 1.61; 95% CI, 1.22-2.14) and difficulty with subspecialty referral (aOR, 1.54; 95% CI, 1.16-2.04) (Table 2). For all children, living in a state with most inclusive policies for health insurance for immigrant children was associated with higher odds of uninterrupted health insurance (aOR, 1.50; 95% CI, 1.32-1.71) and usual place of primary care (aOR, 1.21; 95% CI, 1.07-1.37), but not other outcomes (Table 2). Lower income levels and lower parental educational attainment were significantly associated with decreased odds of uninterrupted health insurance, usual place for primary care, and usual place for sick care, and increased odds of foregone care and difficulty with referrals (Table 2). Driver’s license policies were associated with increased odds of uninterrupted health insurance (aOR, 1.18; 95% CI, 1.07-1.30) but also with increased difficulty with subspecialty referrals (aOR, 1.34; 95% CI, 1.18-1.51). In these multivariable models there were racial and ethnic disparities, with decreased odds of having a usual place for primary care and for sick care for Non-Hispanic American Indian and Alaska Native, non-Hispanic Asian, non-Hispanic Black, Hispanic, and non-Hispanic Native Hawaiian and Other Pacific Islander children compared with Non-Hispanic White children (Table 2).

**Table 2.** Multivariable Logistic Regression Analyses of Primary Outcomes

Characteristic	Outcome, aOR (95% CI)				
	Uninterrupted health insurance^a^	Usual place for primary care^b^	Usual place for sick care^c^	Foregone medical care^d^	Difficulty with subspecialty referral^e^
Immigration status					
Born in US	1 [Reference]	1 [Reference]	1 [Reference]	1 [Reference]	1 [Reference]
Immigrant children	0.48 (0.41-0.56)	0.44 (0.38-0.52)	0.62 (0.55-0.70)	1.61 (1.22-2.14)	1.54 (1.16-2.04)
State-level insurance law inclusivity^f^^,^^g^					
Least inclusive	1 [Reference]	1 [Reference]	1 [Reference]	1 [Reference]	1 [Reference]
Moderately inclusive	0.96 (0.89-1.03)	1.08 (0.99-1.17)	1.00 (0.95-1.05)	0.97 (0.86-1.10)	1.14 (1.01-1.27)
Most inclusive	1.50 (1.32-1.71)	1.21 (1.07-1.37)	0.96 (0.89-1.03)	0.90 (0.76-1.06)	1.03 (0.88-1.21)
State median income (per $10 000 dollar increase)^g^	1.02 (0.99-1.06)	0.96 (0.92-0.99)	0.95 (0.93-0.97)	1.11 (0.98-1.26)	0.96 (0.92-1.01)
Driver’s license for immigrants state policy ^g^	1.18 (1.07-1.30)	0.99 (0.89-1.09)	1.06 (0.99-1.13)	1.10 (0.98-1.26)	1.34 (1.18-1.51)
Age, y					
0-5	1 [Reference]	1 [Reference]	1 [Reference]	1 [Reference]	1 [Reference]
6-11	1.01 (0.92-1.11)	0.99 (0.89-1.10)	1.07 (1.01-1.14)	1.85 (1.58-2.16)	1.31 (1.14-1.51)
12-17	0.92 (0.84-1.01)	0.86 (0.78-0.95)	0.98 (0.92-1.04)	2.34 (2.01-2.73)	1.19 (1.05-1.36)
Male sex	1.06 (0.99-1.15)	1.03 (0.95-1.11)	1.07 (1.02-1.13)	0.96 (0.86-1.07)	1.09 (0.98-1.21)
Race and ethnicity (combined)					
Hispanic	0.74 (0.65-0.84)	0.70 (0.62-0.80)	0.68 (0.63-0.74)	1.27 (1.08-1.50)	1.23 (1.03-1.47)
Non-Hispanic American Indian or Alaska Native	0.47 (0.36-0.60)	0.73 (0.54-0.98)	0.60 (0.48-0.75)	1.15 (0.79-1.69)	1.74 (1.19-2.54)
Non-Hispanic Asian	1.32 (1.08-1.62)	0.54 (0.45-0.64)	0.45 (0.41-0.51)	0.61 (0.44-0.83)	1.12 (0.84-1.49)
Non-Hispanic Black or African American	0.91 (0.81-1.02)	0.72 (0.64-0.81)	0.54 (0.51-0.58)	1.16 (0.96-1.38)	1.04 (0.87-1.25)
Non-Hispanic Native Hawaiian and other Pacific Islander	0.53 (0.28-0.98)	0.70 (0.47-1.04)	0.42 (0.30-0.60)	0.85 (0.39-1.85)	1.88 (0.88-4.01)
Non-Hispanic White	1 [Reference]	1 [Reference]	1 [Reference]	1 [Reference]	1 [Reference]
Other^h^	0.98 (0.86-1.12)	0.98 (0.85-1.14)	0.88 (0.80-0.96)	1.28 (1.07-1.53)	1.12 (0.93-1.35)
Caregiver income, % federal poverty level					
0-199	0.49 (0.43-0.56)	0.53 (0.46-0.61)	0.60 (0.56-0.64)	2.62 (2.19-3.14)	1.65 (1.40-1.93)
200-299	0.50 (0.44-0.57)	0.59 (0.51-0.68)	0.73 (0.67-0.77)	2.06 (1.72-2.46)	1.49 (1.24-1.77)
300-399	0.56 (0.49-0.66)	0.74 (0.63-0.86)	0.78 (0.72-0.85)	1.70 (1.41-2.04)	1.39 (1.17-1.65)
≥400	1 [Reference]	1 [Reference]	1 [Reference]	1 [Reference]	1 [Reference]
Caregiver employment					
At least 1 caregiver employed part time	1 [Reference]	1 [Reference]	1 [Reference]	1 [Reference]	1 [Reference]
No caregiver employed at least part time	0.86 (0.77-0.96)	0.90 (0.80-1.01)	0.83 (0.77-0.89)	1.60 (1.36-1.87)	1.14 (0.97-1.34)
Caregiver highest education					
Less than high school	0.29 (0.25-0.34)	0.27 (0.23-0.31)	0.34 (0.30-0.38)	1.12 (0.87-1.46)	1.43 (1.06-1.93)
High school	0.55 (0.49-0.61)	0.38 (0.34-0.42)	0.40 (0.38-0.43)	0.94 (0.81-1.11)	1.01 (0.85-1.19)
Some college or associate degree	0.67 (0.61-0.74)	0.60 (0.53-0.66)	0.61 (0.57-0.65)	1.16 (1.02-1.33)	1.17 (1.01-1.37)
College degree or higher	1 [Reference]	1 [Reference]	1 [Reference]	1 [Reference]	1 [Reference]
Caregiver immigration status					
At least 1 caregiver born outside US	1.12 (0.98-1.29)	0.85 (0.75-0.97)	0.84 (0.78-0.91)	0.84 (0.70-1.02)	1.00 (0.83-1.19)
Neither born outside US	1 [Reference]	1 [Reference]	1 [Reference]	1 [Reference]	1 [Reference]
Language for survey					
English	1 [Reference]	1 [Reference]	1 [Reference]	1 [Reference]	1 [Reference]
Spanish	0.73 (0.60-0.89)	0.76 (0.63-0.91)	0.94 (0.82-1.06)	0.95 (0.70-1.28)	0.90 (0.67-1.21)
Other	0.40 (0.33-0.48)	0.42 (0.35-0.49)	0.80 (0.71-0.91)	1.06 (0.75-1.50)	1.23 (0.90-1.70)

Abbreviation: aOR, adjusted odds ratio.

^a^ Survey population = 265 837; population estimate = 68 368 685.

^b^ Survey population = 267 064; population estimate = 68 906 244.

^c^ Survey population = 265 846; population estimate = 68 533 186.

^d^ Survey population = 267 925; population estimate = 69 081 520.

^e^ Survey population = 49 864; population estimate = 12 114 799. The subpopulation used only children who were reported to need a subspecialty referral.

^f^ Insurance inclusivity defined as: (1) least inclusive (only certain immigration statuses qualify for insurance and required 5-year waiting period), (2) moderately inclusive (only certain immigration statuses qualify for insurance and no 5-year waiting period), and (3) most inclusive (insurance access for all children regardless of immigration status).

^g^ This categorization varies by year and state.

^h^ Two or more races or any race or ethnicity not otherwise specified.

The interaction term between child immigration status and state health insurance policy was significant (OR, 2.13; 95% CI, 1.38-2.27). Immigrant children demonstrated larger-magnitude associations of living in the most inclusive policy states with having uninterrupted health insurance (aOR, 3.01; 95% CI, 1.89-4.79) compared with US-born children (aOR, 1.44; 95% CI, 1.26-1.65). In the subanalysis with the population restricted to immigrant children, there were increased odds of having a usual place of primary care (aOR, 1.61; 95% CI, 1.07-2.41) in states with the most inclusive policy. There were no statistically significant differences by state-level insurance policy inclusivity for the other outcomes (Table 3). Imputed models had similar results to the complete case analysis models (eTable 4 and eTable 5 in Supplement 1).

**Table 3.** Multivariable Logistic Regression Analysis of Primary Outcomes Restricted to Immigrant Children

Characeristic	Outcome, aOR (95% CI)				
	Uninterrupted health insurance^a^	Usual place for primary care^b^	Usual place for sick care^c^	Foregone medical care^d^	Difficulty with subspecialty referral^e^
**State-level insurance law inclusivity** ^f^ ^,^ ^g^					
Least inclusive	1 [Reference]	1 [Reference]	1 [Reference]	1 [Reference]	1 [Reference]
Moderately inclusive	1.12 (0.82-1.52)	1.19 (0.89-1.57)	0.82 (0.64-1.06)	1.06 (0.61-1.84)	1.12 (0.61-2.04)
Most inclusive	3.01 (1.89-4.79)	1.61 (1.07-2.41)	0.89 (0.64-1.23)	1.28 (0.56-2.90)	1.10 (0.48-2.50)
State median income (per $10 000 dollar increase)^g^	1.05 (0.92-1.20)	1.03 (0.92-1.16)	0.96 (0.89-1.06)	0.89 (0.73-1.10)	1.09 (0.88-1.35)
Driver’s license for immigrants state policy^g^	1.41 (0.99-2.01)	0.88 (0.64-1.22)	1.14 (0.89-1.47)	0.90 (0.50-1.64)	1.02 (0.57-1.86)
Age, y					
0-5	1 [Reference]	1 [Reference]	1 [Reference]	1 [Reference]	1 [Reference]
6-11	1.34 (0.90-1.98)	1.15 (0.77-1.71)	0.91 (0.66-1.26)	1.23 (0.63-2.40)	1.99 (0.94-4.26)
12-17	1.65 (1.12-1.98)	1.26 (0.86-1.86)	0.96 (0.70-1.32)	1.28 (0.69-2.38)	0.99 (0.46-2.10)
Male sex	1.23 (0.93-1.63)	1.05 (0.861 1.35)	1.06 (0.86-1.30)	1.04 (0.64-1.69)	1.08 (0.66-1.75)
Race and ethnicity (combined)					
Hispanic	0.91 (0.48-1.73)	0.94 (0.55-1.60)	0.88 (0.58-1.33)	1.38 (0.70-2.73)	2.63 (1.28-5.50)
Non-Hispanic American Indian or Alaska Native	21.46 (2.40-192.17)	10.74 (1.24-92.92)	1.47 (0.33-6.46)	0.12 (0.01-1.07)	3.11 (0.32-29.88)
Non-Hispanic Asian	0.74 (0.48-1.15)	0.55 (0.34-0.78)	0.49 (0.37-0.65)	0.60 (0.32-1.13)	1.30 (0.71-2.39)
Non-Hispanic Black or African American	0.83 (0.52-1.34)	0.84 (0.55-1.30)	0.71 (0.51-1.00)	1.29 (0.66-2.50)	1.19 (0.49-2.90)
Non-Hispanic Native Hawaiian and other Pacific Islander	0.58 (0.17-2.01)	1.11 (0.37-3.34)	0.79 (0.28-2.26)	0.02 (0.00-0.13)	5.69 (0.43-74.50)
Non-Hispanic White	1 [Reference]	1 [Reference]	1 [Reference]	1 [Reference]	1 [Reference]
Other^h^	1.85 (0.75-4.56)	2.57 (1.31-5.05)	1.19 (0.74-1.94)	0.89 (0.41-1.94)	1.44 (0.49-4.21)
Caregiver income, % federal poverty level					
0-199	0.21 (0.13-0.33)	0.55 (0.34-0.88)	0.51 (0.36-0.72)	3.65 (1.81-7.34)	2.53 (1.38-4.67)
200-299	0.29 (0.18-0.47)	0.60 (0.36-0.99)	0.59 (0.41-0.83)	3.74 (1.79-7.78)	0.88 (0.41-1.86)
300-399	0.59 (0.28-1.23)	0.68 (0.41-1.12)	0.60 (0.41-0.86)	1.49 (0.68-3.27)	0.81 (0.25-2.62)
≥400 and above	1 [Reference]	1 [Reference]	1 [Reference]	1 [Reference]	1 [Reference]
Caregiver employment					
At least 1 caregiver employed part time	1 [Reference]	1 [Reference]	1 [Reference]	1 [Reference]	1 [Reference]
No caregiver employed at least part time	0.63 (0.44-0.90)	0.75 (0.53-1.04)	0.90 (0.68-1.21)	1.98 (1.11-3.53)	1.05 (0.56-1.99)
Caregiver highest education					
Less than high school	0.43 (0.28-0.67)	0.24 (0.16-0.38)	0.22 (0.15-0.33)	1.36 (0.67-2.75)	1.02 (0.42-2.47)
High school	0.54 (0.37-0.78)	0.48 (0.33-0.69)	0.45 (0.33-0.61)	0.56 (0.31-1.04)	0.71 (0.33-1.53)
Some college or associate degree	1.00 (0.68-1.46)	0.57 (0.40-0.81)	0.52 (0.39-0.69)	0.76 (0.45-1.28)	0.58 (0.29-1.15)
College degree or higher	1 [Reference]	1 [Reference]	1 [Reference]	1 [Reference]	1 [Reference]
Caregiver immigration status					
At least 1 caregiver born outside US	0.52 (0.35-0.78)	0.26 (0.16-0.44)	0.42 (0.32-0.56)	0.63 (0.33-1.22)	0.88 (0.50-1.57)
Neither born outside US	1 [Reference]	1 [Reference]	1 [Reference]	1 [Reference]	1 [Reference]
Language for survey					
English	1 [Reference]	1 [Reference]	1 [Reference]	1 [Reference]	1 [Reference]
Spanish	0.69 (0.37-1.28)	0.92 (0.52-1.61)	1.03 (0.68-1.56)	1.15 (0.54-2.44)	1.46 (0.64-3.31)
Other	1.36 (0.92-2.03)	0.89 (0.65-1.23)	1.05 (0.81-1.36)	1.34 (0.71-2.53)	1.75 (0.91-3.38)

Abbreviation: aOR, adjusted odds ratio.

^a^ Survey population = 8320; population estimate = 2 876 581.

^b^ Survey population = 8409; population estimate = 2 919 493.

^c^ Survey population = 8386; population estimate = 2 915 066.

^d^ Survey population = 8449; population estimate = 2 935 968.

^e^ Survey population = 1589; population estimate = 484 948. The subpopulation used only children who were reported to need a subspecialty referral.

^f^ Insurance inclusivity defined as: (1) least inclusive (only certain immigration statuses qualify for insurance and required 5-year waiting period), (2) moderately inclusive (only certain immigration statuses qualify for insurance and no 5-year waiting period), and (3) most inclusive (insurance access for all children regardless of immigration status).

^g^ This categorization varies by year and state.

^h^ More than 2 races or any race or ethnicity not otherwise specified.

## Discussion

In this retrospective cross-sectional study of US children, disparities existed, with worse health insurance and health care access outcomes for immigrant compared with US-born children, even after adjusting for state-level and individual child and caregiver factors. During the study period of 2016 to 2022, there was an overall increase in the proportion of immigrant children who were uninsured, with the peak of greater than 20% occurring in 2021. In contrast, the proportion of uninsured US-born children remained relatively stable at approximately 5%. In the subanalyses focused on immigrant children, living in states with the most inclusive compared with least inclusive health insurance access policies for immigrant children was associated with improved outcomes, with increased odds of uninterrupted health insurance and having a usual place for primary care.

Our study is novel and important, reporting national, contemporary research demonstrating an association of improved access to health insurance and primary care in states with the most inclusive insurance policies for immigrant children, with a larger effect size for immigrant compared with US-born children. In addition, we report significant disparities for immigrant children in access to health insurance and health care across the US over the period from 2016 to 2022, as similarly reported among adults.^15^ Our study did not find consistent associations of state policy for driver’s license with access to health care. This finding suggests this policy may not fully represent the complexity of transportation barriers and may reflect caregivers’ prioritization of their children’s health needs, even with the risk of immigration enforcement if driving. There are complex interconnections of immigration-related policies including health, transportation, work, education, and enforcement, each of which may impact a patient’s decision and ability to seek care.^16^

Previous studies about health care access outcome disparities among immigrant compared with US-born children primarily occurred prior to insurance changes with CHIPRA and before introduction of more inclusive insurance access policies for immigrants.^1,2,3^ While prior studies found a decreased uninsurance rate and increased public insurance coverage after the 5-year waiting period was waived through CHIPRA, these effects waned by 3 years after adoption.^17,18^ In our population of immigrant children, when examining insurance status in the past 12 months based on insurance policy inclusivity, immigrant children in states with the most inclusive policies demonstrated higher rates of adequate insurance (public and private insurance coverage) and the lowest rates of no insurance or gaps in coverage. This finding suggests access to public insurance is important for children and can prevent gaps in insurance coverage.^16^ At the state level, after California expanded Medicaid to include undocumented children, there were decreased rates of uninsurance, increased access to primary care and fewer unmet care needs.^19,20^ The mechanisms by which this takes effect beyond eligibility are not fully understood, including outreach efforts, enrollment facilitation, and administrative barriers, which are important areas for future research. Despite more states having adopted similar policies, data on the impact of these policies using multiyear data has been limited.^5,6^

While for immigrant children there was an association of insurance eligibility with primary care access, statistically significant differences were not observed for other outcomes (sick care, having foregone care, and difficulty with referrals). For these outcomes and those with smaller-magnitude associations, future research with continued longitudinal analysis will be important for a better understanding of associations of state-level policies with these outcomes. It will also be important to further understand barriers to care despite insurance, including health care professional density, availability, transportation challenges, and language barriers.

Among immigrant children, racial and ethnic disparities existed for these health care access outcomes, even after adjusting for state policy, language, and socioeconomic factors, which is consistent with other studies. One study using a national sample of children found disparities in physical health for racially and ethnically minoritized children compared with White children in immigrant families.^21^ Anti-immigrant rhetoric since the mid-2010s has been identified as a factor leading to diminished health care access by caregivers of immigrant children due to fear of being identified as an undocumented immigrant,^16,22^ which may contribute to the disparities found in health care access. Because US immigration policy is historically rooted in structural racism that has impacted health outcomes,^23,24^ efforts to better understand and ultimately mitigate disparities for immigrant children will require enduring and innovative approaches.

To improve health care access outcomes for immigrant children, it will be important to advance more inclusive health insurance policies for these children at the state level. Other policies may contribute to improving health care access and outcomes for US immigrant children. Expanding state Medicaid coverage is one important policy approach to improve health care access to low-income children, regardless of immigration status.^6,20^ At the caregiver level, strategies to improve health literacy and language barriers to accessing health care include the use of patient navigators and resources for families on obtaining insurance and finding care options.^25^ Additionally, state-level 1115 waivers allowing for multiyear continuous Medicaid enrollment may mitigate avoidable gaps in insurance coverage for Medicaid-eligible children.^26^ These policies may be impactful for immigrant families who often experience substantial barriers to enrollment.

### Limitations

Limitations for our study include that as a large database study, there is a risk of misclassification for individual-level variables. Although we included state and individual variables, it is possible there are confounders not accounted for in our models. The NSCH has limited questions about immigration status; thus, we could not analyze the population of undocumented children, who may have the greatest benefit from living in states with the most-inclusive insurance eligibility policies. The NSCH does not provide data on how long the child had lived in the US or whether the child or family was eligible for or enrolled in state insurance programs specifically based on immigration status, which may result in residual confounding. Given our primary outcome was 12-month insurance status, short-term residents may be selected out, thus potentially biasing our analysis toward the null. Immigration-related fears could lead to concerns about stating immigration status; however, this database has been used in multiple studies focused on child immigrant health.^5,6^ With large database studies, missing data are a concern; however, we had complete data for 94% of the population, and models with imputed data showed similar results to the primary analyses. There was a change in the question of employment status (from full-time or not to full-time, part-time, or neither) in 2020. The COVID-19 pandemic occurred during this timeframe, so trends may not be generalizable postpandemic. Because this is a cross-sectional study, we can only cite association and not causation. Nonrandomized control designs could provide stronger evidence, but the dataset does not have sufficient data pre- and postimplementation across enough states to perform this analysis.

## Conclusions

In this cross-sectional study of access to health care, disparities in access to health insurance and health care existed for children who are immigrants. The category of most inclusive state-level insurance eligibility policies was associated with improved access to health insurance and primary care. Advancing inclusive state policies for insurance eligibility may help address health access disparities for immigrant children. Improving access to health insurance and health care will be important to ultimately improve health equity for immigrant children in the US.

## Notes

Supplement 1. eTable 1. Primary Outcomes and Defining Questions From the National Survey of Children’s Health, 2016-2022 eTable 2. State Insurance Eligibility Policy for Immigrant Children, 2016-2022 eTable 3. Health Insurance and Healthcare Access Outcomes for Immigrant and US-Born Children, 2016-2022 eTable 4. Imputed Multivariable Logistic Regression Analysis of Primary Outcomes eTable 5. Imputed Multivariable Analysis of Primary Outcomes Restricted to Immigrant Children

Supplement 2. Data Sharing Statement
