# Supplementary material for: Health Care Access Outcomes for Immigrant Children and State Insurance Policy
Source: JAMA Netw Open. 2025 Dec 1;8(12):e2545826. doi: 10.1001/jamanetworkopen.2025.45826 (PMC12670199; doi:10.1001/jamanetworkopen.2025.45826)
Supplement: Supplement 1. — eTable 1. Primary Outcomes and Defining Questions From the National Survey of Children’s Health, 2016-2022 eTable 2. State Insurance Eligibility Policy for Immigrant Children, 2016-2022 eTable 3. Health Insurance and Healthcare Access Outcomes for Immigrant and US-Born Children, 2016-2022 eTable 4. Imputed Multivariable Logistic Regression Analysis of Primary Outcomes eTable 5. Imputed Multivariable Analysis of Primary Outcomes Restricted to Immigrant Children [file jamanetwopen-e2545826-s001.pdf]

Supplemental Online Content

Douglas KE, Monuteaux MC, Peeler KR, et al. Health care access outcomes for immigrant children and state insurance policy. *JAMA Netw. Open*. 2025;8(12):e2545826. doi:10.1001/jamanetworkopen.2025.45826

eTable 1. Primary Outcomes and Defining Questions From the National Survey of Children's Health, 2016-2022

eTable 2. State Insurance Eligibility Policy for Immigrant Children, 2016-2022

eTable 3. Health Insurance and Healthcare Access Outcomes for Immigrant and US-Born Children, 2016-2022

eTable 4. Imputed Multivariable Logistic Regression Analysis of Primary Outcomes

eTable 5. Imputed Multivariable Analysis of Primary Outcomes Restricted to Immigrant Children

This supplemental material has been provided by the authors to give readers additional information about their work.

© 2025 Douglas KE et al. *JAMA Network Open*.

eTable 1. Primary Outcomes and Defining Questions from the National Survey of Children's Health, 2016-2022

Primary outcome	Defining question in NSCH
1. Uninterrupted insurance access	"Is this child currently covered by any of the following types of health insurance or health coverage plans?"<br>"During the past 12 months, was this child ever covered by any kind of health insurance or health coverage plan?"
2. Usual place of primary care	"Is there a place that this child usually goes when they need routine preventive care, such as a physical examination or well-child check-up?"
3. Usual source of sick care	"Is there a place you or another caregiver usually take this child when they are sick or you need advice about their health?"
4. Foregone healthcare	"During the past 12 months, was there any time when this child needed health care but it was not received?"
5. Difficulty with subspecialty referrals	"During the past 12 months, did this child need a referral to see any doctors or receive any services?" (must have answered yes to be included in subset analysis)<br>"If yes, how much of a problem was it to get referrals?" (2016-2017)<br>"If yes, how difficult was it to get referrals?" (2018-2022)

© 2025 Douglas KE et al. *JAMA Network Open*.

eTable 2. State insurance Eligibility Policy for Immigrant Children, 2016-2022

State	Moderately Inclusive Policy	Year Enacted	Source	Most Inclusive Policy	Year Enacted	Source
Alabama	No	-	https://www.medicaid.gov/medicaid/enrollment-strategies/medicaid-and-chip-coverage-lawfully-residing-children-pregnant-individuals	No	-	
Alaska	No	-	https://www.medicaid.gov/medicaid/enrollment-strategies/medicaid-and-chip-coverage-lawfully-residing-children-pregnant-individuals	No	-	
Arizona	No	-	https://www.medicaid.gov/medicaid/enrollment-strategies/medicaid-and-chip-coverage-lawfully-residing-children-pregnant-individuals	No	-	
Arkansas	Yes	2018	http://www.arkleg.state.ar.us/assembly/2017/2017R/Bills/HCR1012.pdf	No	-	https://www.medicaid.gov/medicaid/enrollment-strategies/medicaid-and-chip-coverage-lawfully-residing-children-pregnant-individuals
California	Yes	2009	https://www.medicaid.gov/medicaid/enrollment-strategies/medicaid-and-chip-coverage-lawfully-residing-children-pregnant-individuals	Yes	2016	https://leginfo.legislature.ca.gov/faces/billNavClient.xhtml?bill_id=201520160SB4
Colorado	Yes	2015	https://www.kff.org/report-section/medicaid-and-chip-eligibility-enrollment-renewal-and-cost-sharing-policies-as-of-january-2016-medicaid-and-chip-eligibility/	Yes	2025	https://leg.colorado.gov/bills/hb22-1289
Connecticut	Yes	2009	https://www.medicaid.gov/medicaid/enrollment-strategies/medicaid-and-chip-coverage-lawfully-residing-children-pregnant-individuals	Yes	2021	https://www.cga.ct.gov/2024/rpt/pdf/2024-R-0040.pdf

© 2025 Douglas KE et al. *JAMA Network Open*.

Delaware	Yes	2011	https://casetext.com/regulation/delaware-administrative-code/title-16-health-and-safety/department-of-health-and-social-services/division-of-social-services/delaware-social-services-manual/medicaid-general-eligibility-requirements/section-14000-14350-legal-immigrant-pregnant-women-and-children-under-age-21#:~:text=Section%2014000%2D14350%20%2D%20Legal%20Immigrant%20Pregnant%20Women,residing%20in%20the%20United%20States%20and%20are	No	-	
District of Columbia	Yes	2009	https://www.medicaid.gov/medicaid/enrollment-strategies/medicaid-and-chip-coverage-lawfully-residing-children-pregnant-individuals	Yes	2010	https://code.dccouncil.gov/us/dc/council/code/sections/1-307.02
Florida	Yes	2017	https://www.flsenate.gov/Session/Bill/2016/89/Analyses/h0089e.HHSC.PDF	No	-	
Georgia	Yes	2024	https://ccf.georgetown.edu/2024/10/15/more-states-expanding-medicaid-chip-for-pregnant-women-including-immigrants/	No	-	
Hawaii	Yes	2009	https://www.medicaid.gov/medicaid/enrollment-strategies/medicaid-and-chip-coverage-lawfully-residing-children-pregnant-individuals	No	-	
Idaho	No	-	https://www.medicaid.gov/medicaid/enrollment-strategies/medicaid-and-chip-coverage-lawfully-residing-children-pregnant-individuals	No	-	
Illinois	Yes	2009	https://www.medicaid.gov/medicaid/enrollment-strategies/medicaid-and-chip-coverage-lawfully-residing-children-pregnant-individuals	Yes	2006	https://www.ilga.gov/legislation/94/HB/09400HB0806sam003.htm

© 2025 Douglas KE et al. *JAMA Network Open*.

Indiana	No	-	https://www.medicaid.gov/medicaid/enrollment-strategies/medicaid-and-chip-coverage-lawfully-residing-children-pregnant-individuals	No	-	
Iowa	Yes	2009	https://www.medicaid.gov/medicaid/enrollment-strategies/medicaid-and-chip-coverage-lawfully-residing-children-pregnant-individuals	No	-	
Kansas	No	-	https://www.medicaid.gov/medicaid/enrollment-strategies/medicaid-and-chip-coverage-lawfully-residing-children-pregnant-individuals	No	-	
Kentucky	Yes	2014	https://www.medicaid.gov/medicaid/enrollment-strategies/medicaid-and-chip-coverage-lawfully-residing-children-pregnant-individuals	No	-	
Louisiana	Yes	2020	https://ldh.la.gov/assets/medicaid/StatePlan/Amend2019/19-0009/19-0009CMSSubmittal.pdf	No	-	
Maine	Yes	2009	https://www.medicaid.gov/medicaid/enrollment-strategies/medicaid-and-chip-coverage-lawfully-residing-children-pregnant-individuals	Yes	2023	http://www.mainelegislature.org/legis/bills/getPDF.asp?paper=HP0156&item=7&snum=130
Maryland	Yes	2009	https://www.medicaid.gov/medicaid/enrollment-strategies/medicaid-and-chip-coverage-lawfully-residing-children-pregnant-individuals	No	-	
Massachusetts	Yes	2009	https://www.medicaid.gov/medicaid/enrollment-strategies/medicaid-and-chip-coverage-lawfully-residing-children-pregnant-individuals	Yes	1997	https://www.mass.gov/doc/all-provider-bulletin-269-amendments-to-all-provider-bulletin-251-0/download
Michigan	Yes	2024	https://legislature.mi.gov/Bills/Bill?ObjectName=2022-HB-6442	No	-	
Minnesota	Yes	2009	https://www.medicaid.gov/medicaid/enrollment-strategies/medicaid-and-chip-coverage-lawfully-residing-children-pregnant-individuals	Yes	2025	https://mn.gov/dhs/assets/minnesotacare-eligibility-for-undocumented-people-faq_tcm1053-653332.pdf

© 2025 Douglas KE et al. *JAMA Network Open*.

Mississippi	No	-	https://www.medicaid.gov/medicaid/enrollment-strategies/medicaid-and-chip-coverage-lawfully-residing-children-pregnant-individuals	No	-	
Missouri	No	-	https://www.medicaid.gov/medicaid/enrollment-strategies/medicaid-and-chip-coverage-lawfully-residing-children-pregnant-individuals	No	-	
Montana	Yes	2009	https://www.medicaid.gov/medicaid/enrollment-strategies/medicaid-and-chip-coverage-lawfully-residing-children-pregnant-individuals	No	-	
Nebraska	Yes	2009	https://www.medicaid.gov/medicaid/enrollment-strategies/medicaid-and-chip-coverage-lawfully-residing-children-pregnant-individuals	No	-	
Nevada	Yes	2019	https://dpbh.nv.gov/uploadedFiles/dpbhnvgov/content/Programs/MIP/Images/SB325info.pdf	No	-	
New Hampshire	Yes	2024	https://ccf.georgetown.edu/2024/10/15/more-states-expanding-medicaid-chip-for-pregnant-women-including-immigrants/	No	-	
New Jersey	Yes	2009	https://www.medicaid.gov/medicaid/enrollment-strategies/medicaid-and-chip-coverage-lawfully-residing-children-pregnant-individuals	Yes	2023	https://pub.njleg.gov/bills/2020/PL21/132_.HTM
New Mexico	Yes	2009	https://www.medicaid.gov/medicaid/enrollment-strategies/medicaid-and-chip-coverage-lawfully-residing-children-pregnant-individuals	No	-	
New York	Yes	2009	https://www.medicaid.gov/medicaid/enrollment-strategies/medicaid-and-chip-coverage-lawfully-residing-children-pregnant-individuals	Yes	1991	https://bplc.cssny.org/pbm/health-programs/child-health-plus/overview
North Carolina	Yes	2009	https://www.medicaid.gov/medicaid/enrollment-strategies/medicaid-and-chip-coverage-lawfully-residing-children-pregnant-individuals	No	-	

© 2025 Douglas KE et al. *JAMA Network Open*.

North Dakota	Yes	2024	https://ccf.georgetown.edu/2024/10/15/more-states-expanding-medicaid-chip-for-pregnant-women-including-immigrants/	No	-	
Ohio	Yes	2014	https://www.registerofohio.state.oh.us/pdfs/5101/1/37/5101$1-37-58_FF_N_RU_20130920_0940.pdf	No	-	
Oklahoma	No	-	https://www.medicaid.gov/medicaid/enrollment-strategies/medicaid-and-chip-coverage-lawfully-residing-children-pregnant-individuals	No	-	
Oregon	Yes	2009	https://www.medicaid.gov/medicaid/enrollment-strategies/medicaid-and-chip-coverage-lawfully-residing-children-pregnant-individuals	Yes	2018	https://www.oregon.gov/oha/hsd/ohp/documents/sb%20558%20frequently%20asked%20questions.pdf
Pennsylvania	Yes	2009	https://www.medicaid.gov/medicaid/enrollment-strategies/medicaid-and-chip-coverage-lawfully-residing-children-pregnant-individuals	No	-	
Rhode Island	Yes	2009	https://www.medicaid.gov/medicaid/enrollment-strategies/medicaid-and-chip-coverage-lawfully-residing-children-pregnant-individuals	Yes	2023	https://trackbill.com/bill/rhode-island-senate-bill-2187-an-act-relating-to-state-affairs-and-government-health-care-for-children-and-pregnant-women-expands-the-rite-track-program-to-provide-health-care-coverage-to-children-up-to-age-nineteen-19-funded-by-federal-funds-if-available-or-if-not-available-by-state-funds/2221450/
South Carolina	Yes	2018	https://www.scdhhs.gov/sites/default/files/documents/(2019-03-04)%20Community%20Engagement%201115%20Demonstration_0.pdf	No	-	
South Dakota	No	-	https://www.medicaid.gov/medicaid/enrollment-strategies/medicaid-and-chip-coverage-lawfully-residing-children-pregnant-individuals	No	-	

© 2025 Douglas KE et al. *JAMA Network Open*.

Tennessee	Yes	2024	https://ccf.georgetown.edu/2024/10/15/more-states-expanding-medicaid-chip-for-pregnant-women-including-immigrants/	No	-	
Texas	Yes	2009	https://www.medicaid.gov/medicaid/enrollment-strategies/medicaid-and-chip-coverage-lawfully-residing-children-pregnant-individuals	No	-	
Utah	Yes	2016	https://le.utah.gov/~2016/bills/static/HB0002.html	Yes	2024	https://le.utah.gov/~2023/bills/static/SB0217.html
Vermont	Yes	2011	https://finance.vermont.gov/sites/finance/files/FIN-FY12_ACT_63.pdf	Yes	2023	https://dvha.vermont.gov/information-for-non-citizens#:~:text=Act%2048%20(H.,IHIP%20launched%20July%201%2C%202022.
Virginia	Yes	2009	https://www.medicaid.gov/medicaid/enrollment-strategies/medicaid-and-chip-coverage-lawfully-residing-children-pregnant-individuals	No	-	
Washington	Yes	2009	https://www.medicaid.gov/medicaid/enrollment-strategies/medicaid-and-chip-coverage-lawfully-residing-children-pregnant-individuals	Yes	1997	https://ccf.georgetown.edu/2009/02/22/washington-state-coverage-to-all-children/
West Virginia	Yes	2015	https://www.kff.org/report-section/modern-era-medicaid-tables/	No	-	
Wisconsin	Yes	2009	https://www.medicaid.gov/medicaid/enrollment-strategies/medicaid-and-chip-coverage-lawfully-residing-children-pregnant-individuals	No	-	
Wyoming	No	-	https://www.medicaid.gov/medicaid/enrollment-strategies/medicaid-and-chip-coverage-lawfully-residing-children-pregnant-individuals	No	-	

© 2025 Douglas KE et al. *JAMA Network Open*.

eTable 3. Health Insurance and Healthcare Access Outcomes for Immigrant and US-Born Children, 2016-2022

	**Immigrant Children*** n (%)	**Born in US*** n (%)
**N**	3,097,329 (4.3%)	69,375,723 (95.7%)
**Health insurance access**		
Public insurance full year	905,840 (30.0%)	19,997,313 (29.3%)
Private insurance full year	1,474,037 (48.8%)	42,475,977 (62.2%)
Unspecified insurance full year	10,770 (0.4%)	152,504 (0.2%)
Uninsured full year	499,215 (16.5%)	3,568,992 (5.2%)
Gaps in coverage	128,466 (4.3%)	2,121,396 (3.1%)
**Usual place of primary care**		
Yes	2,257,199 (73.6%)	63,169,506 (91.7%)
No	808,465 (26.4%)	5,749,052 (8.3%)
**Usual place for sick care**		
Doctor’s office, clinic, health center, or hospital outpatient	1,713,991 (55.9%)	51,229,078 (74.8%)
Urgent care or retail clinic	37,418 (1.2%)	1,261,548 (1.8%)
Emergency department	62,284 (2.0%)	811,128 (1.2%)
Other location	29,025 (0.9%)	447,509 (0.7%)
No usual place	1,220,917 (39.9%)	14,774,140 (21.6%)
**Foregone medical care**		
No	2,925,580 (94.8%)	67,048,571 (97.1%)
Yes	159,800 (5.2%)	2,019,885 (2.9%)
**Difficulty with subspecialty referral**		
No	351,376 (11.4%)	9,594,597 (13.9%)
Yes	155,877 (5.1%)	2,474,734 (3.6%)
Did not need referral	2,570,966 (83.5%)	56,924,264 (82.5%)

*Frequencies are survey-weighted population estimates

© 2025 Douglas KE et al. *JAMA Network Open*.

eTable 4. Imputed Multivariable Logistic Regression Analysis of Primary Outcomes

	Uninterrupted health insurance[1] *aOR (95% CI)*	Usual place for primary care[2] *aOR (95% CI)*	Usual place for sick care[3] *aOR (95% CI)*	Foregone medical care[4] *aOR (95% CI)*	Difficulty with subspecialty referral[5] *aOR (95% CI)*
**Immigration status**					
Born in US	Referent	Referent	Referent	Referent	Referent
Immigrant children	**0.48 (0.41, 0.56)**	**0.45 (0.39, 0.52)**	**0.61 (0.55, 0.68)**	**1.56 (1.18, 2.05)**	**1.50 (1.13, 2.00)**
**State-level insurance law inclusivity[6,7]**					
Least inclusive	Referent	Referent	Referent	Referent	Referent
Moderately inclusive	0.97 (0.90, 1.04)	1.07 (0.99, 1.15)	0.99 (0.94, 1.04)	0.96 (0.87, 1.09)	1.13 (1.01, 1.26)
Most inclusive	**1.54 (1.36, 1.74)**	**1.18 (1.06, 1.33)**	0.95 (0.88, 1.03)	0.90 (0.76, 1.06)	1.01 (0.87, 1.19)
**State median income[7]** (per $10,000 dollar increase)	1.02 (0.98, 1.05)	**0.94 (0.93, 0.98)**	**0.95 (0.93, 0.97)**	**1.01 (0.97, 1.06**)	0.97 (0.92, 1.01)
**Driver's license for immigrants policy[7]** (referent: no driver's license policy)	**1.15 (1.05, 1.27)**	1.02 (0.93, 1.13)	1.07 (1.00, 1.13)	1.11 (0.98, 1.26)	**1.32 (1.17, 1.50)**
**Age** (years)					
0-5	Referent	Referent	Referent	Referent	Referent
6-11	1.02 (0.93, 1.12)	0.99 (0.90, 1.09)	**1.08 (1.01, 1.14)**	**1.93 (1.65, 2.24)**	**1.29 (1.12, 1.48)**
12-17	0.93 (0.85, 1.01)	**0.86 (0.78, 0.95)**	0.98 (0.93, 1.04)	**2.39 (2.06, 2.77)**	**1.18 (1.04, 1.35)**
**Sex** (referent: female)	1.06 (0.99, 1.14)	1.04 (0.97, 1.12)	**1.08 (1.03, 1.13)**	0.98 (0.88, 1.09)	1.09 (0.98, 1.21)
**Race and ethnicity** (combined)					
Hispanic	**0.73 (0.65, 0.83)**	**0.69 (0.61, 0.79)**	**0.65 (0.61, 0.71)**	**1.25 (1.06, 1.47)**	1.20 (1.01, 1.42)
Non-Hispanic American Indian or Alaska Native	**0.47 (0.37, 0.60)**	**0.71 (0.54, 0.95)**	**0.59 (0.48, 0.73)**	1.11 (0.76, 1.60)	**1.79 (1.35, 2.83)**
Non-Hispanic Asian	**1.29 (1.05, 1.57)**	**0.53 (0.45, 0.63)**	**0.46 (0.42, 0.52)**	**0.64 (0.47, 0.87)**	1.10 (0.83, 1.46)
Non-Hispanic Black or African American	**0.89 (0.80, 0.99)**	**0.71 (0.63, 0.78)**	**0.53 (0.50, 0.57)**	1.11 (0.93, 1.33)	1.04 (0.87, 1.23)
Non-Hispanic Native Hawaiian and Other Pacific Islander	0.59 (0.32, 1.09)	**0.62 (0.41, 0.93)**	**0.41 (0.29, 0.57)**	0.80 (0.37, 1.73)	1.72 (0.81, 3.67)
Non-Hispanic White	Referent	Referent	Referent	Referent	Referent
Other	0.98 (0.85, 1.12)	0.96 (0.84, 1.11)	**0.87 (0.80, 0.95)**	**1.31 (1.10, 1.56)**	1.12 (0.93, 1.35)
**Caregiver income**					
0-199% FPL[8]	**0.50 (0.44, 0.57)**	**0.54 (0.47, 0.63)**	**0.59 (0.55, 0.64)**	**2.56 (2.12, 3.08)**	**1.66 (1.42, 1.95)**
200-299% FPL	**0.51 (0.45, 0.58)**	**0.60 (0.52, 0.70)**	**0.72 (0.66, 0.79)**	**2.02 (1.69, 2.40)**	**1.44 (1.22, 1.72)**
300-399% FPL	**0.58 (0.50, 0.67)**	**0.74 (0.64, 0.86)**	**0.79 (0.73, 0.86)**	**1.68 (1.39, 2.04)**	**1.39 (1.18, 1.64)**
400% and above	Referent	Referent	Referent	Referent	Referent
**Caregiver employment**					

© 2025 Douglas KE et al. *JAMA Network Open*.

At least 1 caregiver employed part time	Referent	Referent	Referent	Referent	Referent
No caregiver employed at least part time	**0.86 (0.77, 0.97)**	0.90 (0.81, 1.01)	**0.84 (0.78, 0.91**)	**1.60 (1.37, 1.87)**	1.12 (0.95, 1.32)
**Caregiver highest education**					
Less than high school	**0.29 (0.25, 0.34)**	**0.27 (0.23, 0.31)**	**0.34 (0.31, 0.38)**	1.15 (0.90, 1.47)	1.33 (0.99, 1.78)
High school	**0.53 (0.48, 0.60)**	**0.38 (0.34, 0.42)**	**0.41 (0.38, 0.43)**	0.94 (0.81, 1.10)	1.00 (0.85, 1.18)
Some college or associate degree	**0.66 (0.60, 0.73)**	**0.58 (0.52, 0.64)**	**0.61 (0.58, 0.65)**	**1.19 (1.04, 1.36)**	**1.17 (1.01, 1.36)**
College degree or higher	Referent	Referent	Referent	Referent	Referent
**Caregiver immigration status**					
At least one caregiver born outside US	1.09 (0.95, 1.25)	**0.85 (0.75, 0.96)**	**0.84 (0.78, 0.91)**	0.85 (0.71, 1.02)	1.00 (0.84, 1.20)
Neither born outside US	Referent	Referent	Referent	Referent	Referent
**Language for survey**					
English	Referent	Referent	Referent	Referent	Referent
Spanish	**0.77 (0.64, 0.93)**	**0.77 (0.65, 0.92)**	0.96 (0.85, 1.09)	1.01 (0.75, 1.36)	0.91 (0.68, 1.21)
Other	**0.42 (0.35, 0.51)**	**0.43 (0.37, 0.51)**	**0.79 (0.69, 0.89)**	1.03 (0.73, 1.45)	1.21 (0.89, 1.65)

[1] Survey population 274,131, Population estimate N= 71,288,452
[2] Survey population 275,915, Population estimate N= 72,027,075
[3] Survey population 274,694, Population estimate N= 71,637,158
[4] Survey population 276,760, Population estimate N= 72,185,979
[5] Survey population 51,207, Population estimate N=12,547,119 - Subpopulation used for this analysis of only children who were reported to need a subspecialty referral
[6] Insurance inclusivity defined as: 1) least inclusive (only certain immigration statuses qualify for insurance, required five-year waiting period); 2) moderately inclusive (only certain immigration statuses qualify for insurance, no five-year waiting period); and 3) most inclusive (insurance access for all children regardless of immigration status).
[7] This categorization varies by year and state.
[8] FPL: federal poverty level

© 2025 Douglas KE et al. *JAMA Network Open*.

eTable 5. Imputed Multivariable Analysis of Primary Outcomes Restricted to Immigrant Children

	**Uninterrupted health insurance[1]** *aOR (95% CI)*	**Usual place for primary care[2]** *aOR (95% CI)*	**Usual place for sick care[3]** *aOR (95% CI)*	**Foregone medical care[4]** *aOR (95% CI)*	**Difficulty with subspecialty referral[5]** *aOR (95% CI)*
**State-level insurance law inclusivity[6,7]**					
Least inclusive	Referent	Referent	Referent	Referent	Referent
Moderately inclusive	1.12 (0.83, 1.51)	1.20 (0.91, 1.59)	0.79 (0.62, 1.02)	0.97 (0.57, 1.66)	1.07 (0.59, 1.93)
Most inclusive	**2.93 (1.86, 4.61)**	**1.65 (1.12, 2.44)**	0.84 (0.61, 1.16)	1.31 (0.57, 2.99)	1.03 (0.46, 2.31)
**State median income[7]** (per $10,000 dollar increase)	1.01 (0.89, 1.15)	1.00 (0.89, 1.13)	0.97 (0.88, 1.06)	0.89 (0.72, 1.09)	1.13 (0.91, 1.39)
**Driver's license for immigrants policy[7]** (referent: no driver's license policy)	**1.45 (1.03, 2.03)**	0.91 (0.66, 1.24)	1.17 (0.91, 1.50)	0.84 (0.46, 1.54)	1.02 (0.57, 1.84)
**Age** (years)					
0-5	Referent	Referent	Referent	Referent	Referent
6-11	1.44 (0.98, 2.10)	1.17 (0.80, 1.72)	0.87 (0.63, 1.20)	1.23 (0.63, 2.39)	1.96 (0.94, 4.11)
12-17	**1.70 (1.17, 2.48)**	1.23 (0.85, 1.79)	0.92 (0.67, 1.25)	1.32 (0.72, 2.42)	0.91 (0.43, 1.91)
**Sex** (referent: female)	1.30 (0.98. 1.71)	1.05 (0.82, 1.35)	1.11 (0.91, 1.36)	1.01 (0.63, 1.63)	1.01 (0.62, 1.65)
**Race and ethnicity** (combined)					
Hispanic	0.80 (0.44, 1.46)	1.04 (0.62, 1.75)	0.84 (0.56, 1.24)	1.36 (0.70, 2.67)	**2.72 (1.32, 5.63)**
Non-Hispanic American Indian or Alaska Native	**21.18 (2.36, 190.17)**	**11.49 (1.33, 99.33)**	1.48 (0.33, 6.60)	0.13 (0.01, 1.24)	3.18 (0.33, 30.49)
Non-Hispanic Asian	0.75 (0.49, 1.16)	**0.53 (0.38, 0.74)**	**0.49 (0.37, 0.65)**	0.63 (0.34, 1.18)	1.28 (0.70, 2.35)
Non-Hispanic Black or African American	0.85 (0.53, 1.36)	0.82 (0.54, 1.24)	**0.65 (0.46, 0.91**)	1.25 (0.65, 2.44)	1.10 (0.46, 2.65)
Non-Hispanic Native Hawaiian and Other Pacific Islander	0.63 (0.20, 1.97)	1.13 (0.41, 3.15)	**0.76 (0.29, 2.02)**	**0.01 (0.00, 0.12)**	4.31 (0.39, 47.44)
Non-Hispanic White	Referent	Referent	Referent	Referent	Referent
Other	1.76 (0.74, 4.22)	**2.55 (1.132, 4.91)**	1.18 (0.74, 1.88)	0.91 (0.42, 1.96)	1.37 (0.47, 4.04)
**Caregiver income**					
0-199% FPL[8]	**0.23 (0.15, 0.36)**	**0.58 (0.37, 0.91)**	**0.52 (0.36, 0.75)**	**3.49 (1.78, 6.86)**	**2.55 (1.39, 4.67**)
200-299% FPL	**0.32 (0.19, 0.51)**	**0**.65 (0.40, 1.04)	**0.60 (0.41, 0.87)**	**3.78 (1.84, 7.76)**	0.90 (0.43, 1.88)
300-399% FPL	**0.58 (0.27, 1.25)**	0.71 (0.42, 1.18)	**0.62 (0.43, 0.90)**	1.46 (0.66, 3.21)	0.82 (0.25, 2.71)
400% and above	Referent	Referent	Referent	Referent	Referent
**Caregiver employment**					
At least 1 caregiver employed part time	Referent	Referent	Referent	Referent	Referent

© 2025 Douglas KE et al. *JAMA Network Open*.

No caregiver employed at least part time	**0.67 (0.47, 0.95**)	0.77 (0.52, 1.09)	0.92 (0.68, 1.24)	**1.92 (1.07, 3.42)**	1.00 (0.50, 2.00)
**Caregiver highest education**					
Less than high school	**0.40 (0.26, 0.61)**	**0.24 (0.16, 0.36)**	**0.23 (0.16, 0.33)**	1.31 (0.65, 2.61)	0.99 (0.41, 2.36)
High school	**0.52 (0.36, 0.76)**	**0.45 (0.32, 0.65)**	**0.45 (0.33, 0.61)**	0.60 (0.33, 1.08**)**	0.65 (0.30, 1.41)
Some college or associate degree	0.95 (0.66, 1.38)	**0.53 (0.37, 0.75)**	**0.54 (0.41, 0.72)**	0.75 (0.45, 1.27)	0.61 (0.31, 1.20)
College degree or higher	Referent	Referent	Referent	Referent	Referent
**Caregiver immigration status**					
At least one caregiver born outside US	**0.53 (0.35, 0.81)**	**0.30 (0.18, 0.48)**	**0.46 (0.33, 0.64)**	0.66 (0.34, 1.26)	0.91 (0.52, 1.60)
Neither born outside US	Referent	Referent	Referent	Referent	Referent
**Language for survey**					
English	Referent	Referent	Referent	Referent	Referent
Spanish	0.76 (0.42, 1.36)	0.78 (0.44, 1.35)	1.01 (0.68, 1.49)	1.23 (0.59, 2.57)	1.31 (0.58, 2.97)
Other	1.32 (0.90, 1.95)	0.89 (0.65, 1.23)	1.05 (0.82, 1.36)	1.30 (0.69, 2.44)	1.70 (0.90, 3.24)

[1] Survey population 8,589, Population estimate N=3,008,161

[2] Survey population 8,690, Population estimate N=3,055,678

[3] Survey population 8,669, Population estimate N=3,053,017

[4] Survey population 8,736, Population estimate N=3,074,962

[5] Survey population 1,626, Population estimate N=501,854 - Subpopulation used for this analysis of only children who were reported to need a subspecialty referral

[6] Insurance inclusivity defined as: 1) least inclusive (only certain immigration statuses qualify for insurance, required five-year waiting period); 2) moderately inclusive (only certain immigration statuses qualify for insurance, no five-year waiting period); and 3) most inclusive (insurance access for all children regardless of immigration status).

[7] This categorization varies by year and state.

[8] FPL: federal poverty level

© 2025 Douglas KE et al. *JAMA Network Open*.
