# Supplementary material for: Health Care Access Outcomes for Immigrant Children and State Insurance Policy
Source: JAMA Netw Open. 2025 Dec 1;8(12):e2545826. doi: 10.1001/jamanetworkopen.2025.45826 (PMC12670199; doi:10.1001/jamanetworkopen.2025.45826)
Supplement: Supplement 2. — Data Sharing Statement [file jamanetwopen-e2545826-s002.pdf]

Data Sharing Statement

Douglas. Health Care Access Outcomes for Immigrant Children and State Insurance Policy. *JAMA Netw Open*. Published December 01, 2025. doi:10.1001/jamanetworkopen.2025.45826

Data
Data available: Yes
Data types: Deidentified participant data
How to access data: Through the National Survey of Children's Health data center
https://www.census.gov/programs-surveys/nsch/data/datasets.html
When available: With publication

Supporting Documents
Document types: None

Additional Information
Who can access the data: N/A
Types of analyses: N/A
Mechanisms of data availability: N/A
